# A System of Surgery

**Published:** 1896-09

**Authors:** 


### Book Reviews.

A System of Surgery. By American authors. Edited by Frederic S. Dennis, M. D., Professor of the Principles and Practice of Surgery, Bellevue Hospital Medical College, New York ; President of the American Surgical Association, etc., assisted by John S. Billings, M. D., LL. D., D. C. L., Deputy Surgeon-General, U. S. A. Complete in four imperial octavo volumes, containing 3,652 pages, with index. 1,585 engravings and forty-five full-page plates in black and colors. Volume IV., 970 pages, 441 engravings and twenty-three plates. Price, per volume, $6.00 in cloth ; $7.00 in leather ; $8.50 in half Morocco, gilt back and top. For sale by subscription. Philadelphia : Lea Brothers & Co. 1896.

This volume completes Dennis's system of surgery and is in every way quite up to the standard of excellence which characterizes the preceding volumes. The subjects include tumors, hernia, the surgery of the alimentary canal and of some of the abdominal viscera, the breast, and as evidence of the progressive character of the work a final chapter is devoted to the use of the Röntgen rays in surgery.

The chapter on tumors by the editor is an excellent one. According to the author the development of tumors seems to be due to changes of an inflammatory nature in a large majority of cases, and that mechanical injury and peripheral irritation are the common causes of the preliminary inflammation. As the author puts it, "mechanical injury is associated with sarcoma, notably of bone ; peripheral irritation is connected with carcinoma, and sinus or other irritation is associated with epithelioma." Heredity also plays an important part etiologically, from 12 to 15 per cent. of cases being traceable to this influence.

The pages devoted to the treatment of tumors, particularly those of malignant type, are full of interest, clinical experience and hope. The author's views are stated as follows :

The clinical evidence is such that the surgeon cannot disregard the strong arguments in favor of the reversion to primary cells, induced perhaps by inflammatory causes ; and if this theory is accepted, the local origin of tumors seems evident. The general infection spreads from a local one, and the early and radical removal of the primary focus will prevent the widespread dissemination of malignant disease."

In accordance with this belief early and radical operation is earnestly advised in the treatment of malignant neoplasms. Should the tumor recur, it must be removed again and operation persisted in until final recovery is effected, or the case becomes clearly inoperable. Of the toxin treatment, as practised by Coley, the author has much to say in the way of commendation. While not committing himself to an exact opinion he regards the procedure justifiable and one which offers encouragement for the future.

The chapter upon hernia by Bull and Coley is quite complete. The anatomy and pathology, the various forms and the recognised methods of treatment are all compactly but clearly described. The mortality in 5,000 cases of reducible hernia, treated by some form of radical operation, is given $1\frac{1}{2}$ per cent. From 60 to 90 per cent. remain sound for a number of years. Bassini's operation is preferred by the authors, who have personally operated upon upward of 300 cases with three deaths and seven relapses, all slight. The remaining chapters of the volume are of the same high character and deserve careful reading. Indeed, it is difficult to select when all have such uniform and general excellence.

The publishers have done just what might have been expected and given out a most attractive volume. The type is admirable, the illustrations are clear and most of them new. A few typographical errors such as firbo-lipoma for fibro-lipoma, page 857 ; Mondé for Mundé, page 687, mar, but in no wise affect the great scientific value of the work. J. P.

